# Clinical and Virologic Outcomes of Topical Imiquimod in Vaginal Intraepithelial Neoplasia: A Retrospective Cohort Study

**DOI:** 10.3390/jcm15072499

**Published:** 2026-03-25

**Authors:** Nazlı Aylin Vural, Merve Aldıkaçtıoğlu Talmaç, İzel Günay, İlkbal Temel Yüksel, Gazi Güner, Emel Canaz, Hasan Turan, Baki Erdem, Nilüfer Çetinkaya Kocadal

**Affiliations:** 1 Department of Gynecologic Oncology, Yozgat State Hospital, 66100 Yozgat, Turkey; 2 Molecular Oncology (Master), Graduate School of Education, Istinye University, 34396 Istanbul, Turkey; drmrve@hotmail.com; 3 Department of Gynecologic Oncology, Kanuni Sultan Suleyman Training and Research Hospital, Health Sciences University, 34668 Istanbul, Turkey; 4 Department of Obstetrics & Gynecology, Basaksehir Cam and Sakura State Hospital, Health Sciences University, 34668 Istanbul, Turkey; zel.gunay@gmail.com; 5 Department of Gynecologic Oncology, Basaksehir Cam and Sakura State Hospital, Health Sciences University, 34668 Istanbul, Turkey; drilkbaltemel@gmail.com (İ.T.Y.); dr.gaziguner01@gmail.com (G.G.); 6 Department of Gynecology with Center for Oncological Surgery, European Comprehensive Center for Ovarian Cancer, Charité Medical University of Berlin, 10117 Berlin, Germany; emel.canaz@charite.de; 7 Department of Obstetrics and Gynecology, Mersin City Hospital, University of Health Sciences, 33240 Mersin, Turkey; 8 Department of Obstetrics & Gynecology, Acıbadem University Hospital, 34140 Istanbul, Turkey; 9 Gynecologic Oncology, Department of Obstetrics and Gynecology, Yeditepe University, 34755 Istanbul, Turkey; cetinkayanilufer@gmail.com

**Keywords:** vaginal intraepithelial neoplasia, imiquimod, topical therapy, human papillomavirus

## Abstract

**Purpose**: To describe clinical and virologic outcomes after topical 5% imiquimod for VaIN (vaginal intraepithelial neoplasia) within routine clinical practice, including the assessment of available post-treatment high-risk HPV status, and to evaluate its relationship with treatment outcomes. **Methods**: In this retrospective-cohort study, twenty patients with histologically confirmed VaIN (low-grade VaIN = 10, high-grade VaIN = 10) were evaluated between October 2020 and October 2022. The treatment varied based on the initial VaIN grade. The primary endpoint was complete response, defined as negative cytology and/or benign follow-up biopsy. Secondary endpoints included partial response (downgrading from high-grade to low-grade), persistence/progression, adverse events, and available post-treatment high-risk HPV status. **Results**: Among seventeen evaluable patients, a complete response was observed in 69.2% (9/13) imiquimod-treated patients. In high-grade VaIN imiquimod-treated patients, complete response was observed in 66.7% (6/9), while 33.3% (3/9) downgraded to low-grade VaIN on biopsy as a secondary outcome, with no persistence over a median follow-up of 24 months. Low-grade VaIN showed 75% clearance with either imiquimod or observation at a median follow-up of 24 months. In one low-grade imiquimod-treated, persistent HPV16 was observed, and the lesion progressed to high-grade VaIN; however, no invasive cancers were observed during follow-up. Adverse events related to imiquimod were mild; there were no discontinuations. Among baseline high-risk HPV positive imiquimod-treated patients, post-treatment follow-up HPV testing was available in a limited subset group, and clearance was confirmed in 44% (4/9) among those with follow-up testing and presented descriptively as exploratory. **Conclusions**: In this single-center retrospective cohort, outcomes after topical 5% imiquimod are reported as observed findings. Complete and partial responses were observed in high-grade VaIN with acceptable tolerability and no invasive events at available intermediate follow-up (median 24 months). Post-treatment HPV testing provided complementary virologic information alongside clinical outcomes, consistent with recommendations incorporating HPV testing into VaIN follow-up, and longer surveillance is required to assess long-term oncologic outcomes.

## 1. Introduction

Vaginal intraepithelial neoplasia is an uncommon Human Papillomavirus (HPV) related premalignant lesion within the lower genital tract [1]. Its reported prevalence is approximately 2–3 per 10,000 women, and the estimated incidence ranges from 0.2 to 2 per 100,000 woman-years; furthermore, it represents only 0.4% of premalignant lesions of the lower genital tract [2,3,4]. VaIN is diagnosed much less frequently than cervical intraepithelial neoplasia (CIN) [2,3]. A large cohort study revealed that VaIN was associated with a high risk of recurrence [5], while the risk of progression to vaginal cancer is relatively low, ranging between 2% and 12% [6,7,8]. After the first preinvasive vaginal case was documented, Hummer et al. published a review of treated patients with carcinoma in situ of the vagina at Mayo Clinic [9]. Vaginal intraepithelial neoplasia is currently classified as grade 1 as low-grade vaginal dysplasia and grade 2–3 as high-grade vaginal dysplasia [10]. VaIN is usually asymptomatic, and it is often diagnosed following abnormal cytology or histopathologic evaluation, resulting in further assessment [11]. A higher risk of VaIN has been described in association with persistent high-risk HPV infection and prior high-grade cervical pathology [12]. Among women following hysterectomy, high-grade VaIN has been reported more frequently in those who underwent hysterectomy for CIN3 compared to those treated for benign indications, and high-grade VaIN cases clustered in those with persistent HPV, most commonly involving genotype HPV16 [13,14].

The management of vaginal intraepithelial neoplasia depends on several factors, including the characteristics of the lesion itself (such as grade, extension, and multifocality), as well as patient factors (such as age and sexual activity) [15]. Management options include excision, ablation, and topical therapies [15]. Recent consensus recommendations support follow-up for low-grade vaginal neoplasia and treatment for high-grade vaginal neoplasia, with excision recommended when invasion cannot be ruled out [15].

Imiquimod 5% cream, an immunomodulatory agent and toll-like receptor 7 agonist that stimulates the production of interferon-α and tumor necrosis factor-α [16,17], has been used as a topical, non-surgical intervention for vaginal intraepithelial neoplasia [17,18]. Its utilization as a topical therapy is progressively increasing owing to its ability to act as an immune-response modifier [19,20].

Systematic reviews and meta-analyses suggest the use of imiquimod in VaIN [4,21], and although its use in vaginal intraepithelial neoplasia is increasing, real-world data remain limited regarding patient selection, clinical outcomes, and post-treatment high-risk HPV patterns. We therefore conducted a retrospective cohort study to describe clinical outcomes of vaginal neoplasia managed with topical imiquimod and observation-only approaches, and to summarize post-treatment high-risk HPV findings alongside treatment outcomes in available patients.

## 2. Subjects and Methods

### 2.1. Ethics Approval and Consent

The study was approved by the Local Ethical Committee of Başakşehir Çam and Sakura City Hospital of Health Sciences University (approval number: 2023.05.185). Due to the retrospective design of the study, the requirement for individual informed consent was waived. The study was conducted in accordance with the principles of the Declaration of Helsinki. This manuscript adheres to the Strengthening the Reporting of Observational Studies in Epidemiology (STROBE) guidelines for cohort studies.

### 2.2. Study Design

This single-center retrospective cohort study was conducted at the tertiary referral hospital, Gynecologic Oncological Surgery Clinic (Istanbul, Turkey) between October 2020 and October 2022. The study aimed to describe clinical outcomes following topical 5% imiquimod in patients diagnosed with VaIN, and to explore the relationship between available post-treatment high-risk HPV results and treatment findings.

### 2.3. Study Subjects and Variables

Demographic and clinicopathological data for patients who met the inclusion criteria were retrieved from their medical records. The following variables were included in the study: age, gravidity, parity, smoking status, history of cervical dysplasia, results of the Pap smear cytology test, HPV positivity and genotyping, colposcopy findings, and vaginal biopsy pathology. Information regarding prior hysterectomies, including the indication, if applicable, was also collected. Additionally, the preferred treatment modality and treatment duration, follow-up time, and follow-up biopsy results were recorded.

We reviewed patients diagnosed with vaginal dysplasia based on colposcopic vaginal biopsies performed for various clinical indications. The patients were referred with abnormal Papanicolaou (Pap) smear results and/or HPV positivity for colposcopic examination. After biopsies from suspicious areas, the diagnosis was histologically confirmed as vaginal intraepithelial neoplasia between October 2020 and October 2022.

### 2.4. Intervention and Follow-Up

At the time of diagnosis, patients were counseled on management options, including observation, excision, ablation/laser, and topical therapies, as well as on the rarity of the disease; shared decision-making was emphasized. Recent consensus recommendations support follow-up for low-grade neoplasia and treatment for high-grade neoplasia, with excision recommended when invasion cannot be excluded [14]. It was explained that medical therapy, such as imiquimod 5% cream, might not be sufficient as the sole treatment and that there remains an ongoing risk of recurrence even with treatment. Treatment decisions were made through shared decision-making after counseling on available options, noting that treatment allocation was not randomized and was driven by patient preference and clinical factors. Overall, all patients diagnosed with high-grade vaginal intraepithelial neoplasia initially preferred medical therapy over surgical interventions. Although surveillance is generally recommended for low-grade dysplasia, treatment was offered to select low-grade neoplasia cases due to persistent or multifocal disease, extensive vaginal involvement, high-risk HPV persistence, prior cervical neoplasia, or patient preference.

All imiquimod treatments were administered by Gyn Oncology subspecialists in the clinic. The clinician applied a single sachet of 5% imiquimod cream (equivalent to 12.5 mg) to the vaginal lesions using a sterile swab and waited for a while after application, following the insertion of a speculum into the vagina. This was repeated twice a week for 6 to 8 weeks. In routine practice, frequency (2–3 times weekly) and duration (up to 12 weeks) were individualized based on tolerability and clinical response. Treatment methods included: (i) topical 5% imiquimod cream as detailed above, (ii) conservative management with serial Pap smears and colposcopy, and in one case, (iii) primary surgical excision of high-grade vaginal intraepithelial neoplasia. Follow-up included cytology and colposcopy as clinically indicated; abnormal cytology and/or positive HPV results resulted in colposcopy and biopsy. The first post-treatment evaluation was typically performed approximately 6 months after treatment. Additional biopsies were performed if vaginal cytology revealed abnormalities. During follow-up, further treatment was provided if the condition persisted or recurred.

### 2.5. Outcome Definitions

The primary assessment population consisted of high-grade VaIN patients; although low-grade vaginal neoplasia outcomes were reported separately as supportive descriptive data, given the potential for spontaneous regression. Clinical outcomes were defined on follow-up biopsy: complete response (CR) as negative cytology and/or a benign follow-up biopsy, partial response (PR) as a downgrade from high-grade neoplasia to low-grade neoplasia on biopsy and considered as a secondary outcome rather than treatment success, persistence as unchanged histologic grade, and progression as an upgrade from low-grade neoplasia to high-grade neoplasia or to invasive disease. For relapse evaluation, at least two follow-up visits were required. We only considered a lesion as ‘recurred’ if it had cleared and then was found again on a subsequent follow-up visit.

### 2.6. Statistical Analyses

Statistical analyses were performed using Statistical Package for the Social Sciences (SPSS) software, version 30.0 (IBM Corp., Armonk, NY, USA). Continuous variables were presented as mean ± SD, or median (IQR) as appropriate, and categorical variables were summarized as frequencies and percentages (*n*/*N*, (%)). Given the small sample size due to the rarity of vaginal intraepithelial neoplasia and non-randomized treatment allocation, analyses were primarily descriptive. Treatment outcomes, including complete response, partial response, persistence, and progression, were evaluated descriptively. HPV status before and after treatment was also evaluated descriptively in relation to treatment outcomes. Where exploratory comparisons were performed, Fisher’s exact test was used for categorical variables and the Mann–Whitney U test for continuous variables.

## 3. Results

A total of 20 patients with histologically confirmed vaginal intraepithelial neoplasia, between October 2020 and October 2022, were included in the study; 10 were diagnosed with low-grade, and 10 with high-grade. The median age was 48 years (range, 27–71), and the mean ages were similar in low-grade and high-grade groups (49.9 vs. 48.0 years, respectively). The mean gravidity was 2.2 in the low-grade neoplasia group and 2.9 in the high-grade neoplasia group. Smoking was reported in 3 patients within each group. Baseline patient data are detailed in Table 1.

Fifteen patients (75%) underwent hysterectomy before diagnosis. Prior hysterectomy was commonly performed for cervical neoplasia and endometrial pathology (Table 1). Pre-treatment cervical cytology findings varied across groups, with a negative for intraepithelial lesion or malignancy (NILM) and atypical squamous cells of undetermined significance (ASCUS) representing the most frequent results (Table 1).

High-risk HPV was detected in 16 of 19 patients with available genotyping data. Non-16/18 HPV types were more frequent than HPV16 or HPV18 and more common in high-grade cases than low-grade cases (70% vs. 40%, respectively) (Table 1).

The colposcopic examination identified lesions that were primarily situated in the proximal third of the vagina(*n* = 16; 80%), and multifocal involvement was observed in 40% (*n* = 8) of the cohort, which were more frequent in high-grade neoplasia cases.

Topical imiquimod was utilized as a treatment modality, with a median treatment duration of 6.9 weeks (5% cream, administered as a single sachet containing 12.5 mg, twice or three times weekly, for a range of 6 to 12 weeks) (Table 2). All high-grade patients who were evaluable for the study (*n* = 9/9; 100%) received topical imiquimod, while 50% (*n* = 4/8) of evaluable low-grade patients received imiquimod, with the remaining 50% (*n* = 4/8) managed conservatively through observation alone. In one patient who underwent surgical excision with high-grade vaginal intraepithelial neoplasia, additional topical imiquimod was administered following surgical excision after a 5-month period, due to the persistent high grade in the control biopsy. In one patient who followed through observation, topical imiquimod treatment was administered due to the diagnosis of persistent low-grade vaginal intraepithelial neoplasia after an 11-week period. Following the diagnosis, one patient with low-grade vaginal intraepithelial neoplasia, who was planned for observation, and one patient with high-grade vaginal intraepithelial neoplasia, who was planned for surgical excision, did not revisit the clinic for treatment.

Among patients with high-grade who were treated medically (*n* = 9), complete response was achieved in 66.7% (*n* = 6/9) of patients, while 33.3% (*n* = 3/9) of patients revealed partial regression to low-grade as a secondary outcome (Table 3). No patient with high-grade progressed or exhibited persistent high-grade disease following medical treatment. In low-grade neoplasia patients receiving imiquimod (*n* = 4), complete clearance occurred in 75% (*n* = 3/4), while one patient (25%) progressed to high-grade despite treatment. 

Given the potential for spontaneous regression in low-grade VaIN, these low-grade observation outcomes are reported descriptively and are not used to discuss the treatment effect. In the conservative observation-only low-grade dysplasia cohort (*n* = 4), spontaneous histologic regression to normal intraepithelial morphology was observed in 3 patients (75%), whereas one patient (25%) had persistent low-grade neoplasia, with no evidence of disease progression during follow-up.

The adverse effects associated with the use of topical imiquimod were mild and included transient pruritus in 3 patients (21.4%), vaginal discharge in 1 patient (7.1%), and fever in 1 patient (7.1%). No severe systemic adverse effects occurred, and no treatment discontinuation was required.

The median follow-up duration for evaluable patients who received topical imiquimod treatment (*n* = 13) and had at least two follow-up visits was 24 months, with a range of 10 to 35 months. In this group, patients with high-grade vaginal intraepithelial neoplasia (*n* = 6) had a median follow-up duration of 24 months, with a range of 15 to 33 months, while those with low-grade lesions (*n* = 3) had a median follow-up duration of 14 months, ranging from 10 to 35 months. In the observation-only group (*n* = 4), the median follow-up duration was 24.5 months, with a range of 18 to 26 months. Four of the 17 evaluable patients were lost to follow-up after their first post-treatment visit, so their longer-term outcomes and relapse evaluations are unknown. Among patients lost to long-term follow-up, initial post-treatment assessments demonstrated histologic clearance or regression. One patient with a low-grade lesion who had been planned for observation died of a malignant melanoma before the first follow-up visit.

During this follow-up period, recurrence was noted in one case initially diagnosed as vaginal intraepithelial neoplasia grade 2. This patient achieved clearance with topical imiquimod therapy but subsequently relapsed cytologically as low-grade at 11 months post-treatment. Among patients with low-grade lesions, only one individual progressed to high-grade disease following the initial course of 5% imiquimod treatment. This patient subsequently required an additional course of therapy. In patients diagnosed with high-grade lesions who achieved remission post-treatment, one patient necessitated two courses during the treatment period, while another required three courses of topical 5% imiquimod therapy. Notably, no patients in either the imiquimod or observation cohort progressed to invasive carcinoma.

Post-treatment HPV data were available for 9 of 12 imiquimod-treated patients who were HPV-positive at baseline and were presented descriptively. Given the limited sample size and incomplete testing, no formal statistical testing was performed, and the findings are exploratory. Clearance of HPV occurred in 4 (44.4%) patients (3 with non-16/18 HPV and 1 with HPV 18). Two patients with baseline non-16/18 HPV positivity remained non-16/18 HPV positive, and two patients with baseline HPV16 positivity became negative for HPV 16 but tested positive for non-16/18 HPV types. Notably, the only patient who progressed from low-grade to high-grade had persistent HPV16 positivity at follow-up.

The median time to achieve a negative intraepithelial cytology among ten of the thirteen patients treated with topical 5% imiquimod was 10.5 months (range, 2–24); of these, one patient exhibited recurrence after achieving clearance.

## 4. Discussion

In this single-center retrospective cohort with shared decision-making between physician and patient, we report observed clinical outcomes after topical 5% imiquimod, together with exploratory reporting of available post-treatment high-risk HPV results. Nine patients (69.2%) achieved a complete response on follow-up biopsy, and no invasive cancers were observed during the available follow-up. In the primary high-grade VaIN patients treated with imiquimod (*n* = 9), complete response was observed in 6 (66.7%) patients, while regression from high-grade to low-grade disease was documented in 3 of 9 (33.3%) patients, which is reported as a secondary outcome rather than treatment success.

Considering the potential for spontaneous regression in low-grade VaIN, the outcomes are summarized descriptively, and no definitive conclusions regarding the efficacy of treatment can be drawn. Among patients with low-grade VaIN treated with imiquimod (*n* = 4), complete clearance was observed in 3 (75%) individuals, while 1 (25%) patient progressed to high-grade VaIN during the follow-up period. In the conservative observation-only group, 3 out of 4 patients with low-grade demonstrated spontaneous histologic regression to a negative for intraepithelial lesion or malignancy (NILM) during follow-up, while one patient exhibited persistence. Throughout the available follow-up period, no cases of invasive vaginal carcinoma were detected within the median of 24 months, ranging from 10 to 35 months. These findings contribute valuable data to routine-practice treatment outcomes data and the reporting of cytology and histology results alongside available post-treatment high-risk HPV findings in the surveillance of vaginal intraepithelial neoplasia, emphasizing the importance of recurrence risk assessment and long-term monitoring in management strategies. Nevertheless, these results should not be interpreted as evidence of comparative effectiveness.

Post-treatment high-risk HPV follow-up was available for 9 of the 12 patients with baseline high-risk HPV positivity and is reported descriptively. Among these patients, clearance was documented in 4 of 9 (44.4%), with 3 cases involving non-16/18 HPV and 1 case involving HPV 18. This finding may align with the proposed mechanism whereby imiquimod acts as a toll-like receptor 7 agonist, thereby enhancing local immune surveillance and promoting viral clearance. Furthermore, two cases of non-16/18 HPV persisted, while two patients converted from HPV 16 to non-16/18 HPV. Among those with follow-up testing, persistent high-risk HPV and genotype alterations were observed; however, the sample size was insufficient for statistically significant evaluation. Regarding histologic findings, all patients with high-grade treated with imiquimod showed regression, either complete or partial. The only histologic progression in the study occurred in a patient with low-grade dysplasia who was HPV 16 positive and showed persistence. In the observation-only group, histologic persistence co-occurred with HPV 18 positivity in the single persistent case. Given the small sample size and incomplete HPV follow-up, the observed relationship between high-risk HPV clearance or persistence and clinical outcomes is exploratory, should be interpreted descriptively, and warrants confirmation in future prospective studies.

Our findings generally align with previous studies demonstrating the efficacy and safety of topical 5% imiquimod in the treatment of vaginal intraepithelial neoplasia; however, differences in regimens, outcome definitions, and follow-up across studies limit direct comparisons within the literature [17,18,21,22]. A randomized trial by Tainio et al. reported regression rates of approximately 80% in patients treated with imiquimod [22]. Similarly, prior studies reported complete regression in 57–86% of cases [17,18,23]. In a retrospective study by Field et al., of 29 evaluable patients with low-grade dysplasia managed conservatively, the success rate was 97%, and the recurrence rate was 14% [24]. The TOPIC-3 study, a non-randomized multicenter trial, showed 69% high-risk HPV clearance in the imiquimod group [18]. Consistent with Kim et al., who reported that 96.9% of vaginal intraepithelial neoplasia lesions were located in the upper vagina [7], we also found that 80% of lesions were confined to the upper vaginal region. The upper one-third of the vagina and the cervix originate from the same embryological origin and share similar epithelial characteristics, which may partially explain the heightened susceptibility of this area to HPV-associated neoplastic transformation, especially in patients with a history of cervical intraepithelial neoplasia or carcinoma [10]. These findings underscore the prevalence of dysplasia in the upper vagina and highlight the necessity for meticulous examination of this region during colposcopic assessment to prevent misdiagnosis.

### Strengths and Limitations

Strengths of this study include providing real-world clinical data from a tertiary gynecologic oncology center regarding the management of a rare disease entity. Histologic outcomes were assessed using cytology and/or hystology-confirmed results, allowing objective evaluation of disease response. In addition, the study incorporated post-treatment high-risk HPV testing in available patients, providing complementary virologic outcomes alongside histologic findings.

Limitations include the retrospective single-center study design and shared-decision-making treatment selection without randomization, which introduces selection bias and does not support direct comparison between management approaches as an effectiveness study. In addition, the sample size was relatively small, reflecting the rarity of vaginal intraepithelial neoplasia, which limits the statistical power and the subgroup analyses. Furthermore, follow-up HPV testing and long-term clinical follow-up were not available for all patients, which may limit the assessment of recurrence patterns and virologic clearance. The absence of a randomized comparison group prevents direct comparison between treatment modalities. Finally, the follow-up duration limits conclusions regarding long-term oncologic outcomes, and continued surveillance remains essential.

Future prospective multicenter studies with larger cohorts and longer follow-up duration are needed to better define optimal management strategies and to confirm the role of HPV status in post-treatment surveillance of vaginal intraepithelial neoplasia.

Consistent with guidelines issued by the European Society of Gynecological Oncology (ESGO) [15], the application of this therapeutic approach appears appropriate for carefully selected patients, as it preserves tissue integrity and allows for comprehensive coverage of the vaginal mucosa. Consensus recommendations incorporate HPV testing into follow-up and note that negative co-testing may indicate clearance; accordingly, high-risk HPV status can be considered alongside cytology and biopsy findings during surveillance. Long-term, individualized follow-up is likely necessary to observe potential recurrence, progression, or persistence of vaginal intraepithelial neoplasia. No invasive carcinoma was observed within the available follow-up; however, the follow-up duration and sample size limit conclusions regarding long-term cancer risk, and continued surveillance remains essential. Given the rarity of the disease, multi-institutional studies or meta-analyses will be needed to confirm the role of HPV persistence in post-treatment surveillance and determine optimal management algorithms.

## 5. Conclusions

Overall, imiquimod may be a feasible non-surgical treatment option for carefully selected patients with vaginal intraepithelial neoplasia, provided that invasion has been excluded. Reporting post-treatment high-risk HPV results alongside treatment outcomes may provide complementary information within an HPV-integrated follow-up approach.

## Figures and Tables

**Table 1:** Baseline clinicopathologic characteristics and observed outcomes of the patients.

Case	Age	G/P	Smoking	Previous Cervical Neoplasia	Prior Hysterectomy (If Yes, the Indication)	Referral Cytology	Referral HPV Status	Lesion Localization	Histology at Diagnosis	Treatment Modality	Final Response to Treatment	Follow-Up Period *
Case 1	37	0/0	no	unknown	no	negative	negative	multifocal distal 1/3	High-grade VaIN (VaIN 2/3)	Topical agent (imiquimod)	PR	2 months *
Case 2	61	4/3	no	CIN 1	yes (cervical dysplasia)	ASC-H	HPV 16	unifocal proximal 1/3	High-grade VaIN (VaIN 2)	Topical agent (imiquimod)	PR	33 months
Case 3	46	0/0	yes	CIN 3	yes (cervical dysplasia)	negative	non-16/18 hr HPV	unifocal proximal 1/3	High-grade VaIN (VaIN 2/3)	Topical agent (imiquimod)	CR	15 months
Case 4	71	7/6	no	no	yes (endometrial cancer)	ASC-H	non-16/18 hr HPV	unifocal proximal 1/3	High-grade VaIN (VaIN 2/3)	Topical agent (imiquimod)	CR	24 months
Case 5	50	2/2	no	cervical cancer	yes (cervical cancer)	ASCUS	non-16/18 hr HPV	multifocal proximal 1/3	Low-grade VaIN (VaIN 1)	Observation ^a^ Topical agent ^b^ (imiquimod)	CR	35 months
Case 6	48	3/2	-	no	yes (myoma uteri)	negative	non-16/18 hr HPV	multifocal proximal 1/3	High-grade VaIN (VaIN 2/3)	Surgical excision ^a^ Topical agent ^b^ (imiquimod)	CR	25 months
Case 7	43	1/1	yes	CIN 3	yes (cervical dysplasia)	LSIL	non-16/18 hr HPV	unifocal proximal 1/3	Low-grade VaIN (VaIN 1)	Topical agent (imiquimod)	CR	10 months *
Case 8	60	2/2	no	CIN 3	yes (cervical dysplasia)	LSIL	non-16/18 hr HPV	unifocal proximal 1/3	Low-grade VaIN (VaIN 1)	Observation	CR	23 months
Case 9	34	0/0	yes	CIN 1	no	ASCUS	HPV 18	unifocal middle 1/3	Low-grade VaIN (VaIN 1)	Observation	Persistance	26 months
Case 10	43	4/3	yes	CIN 3	yes (cervical dysplasia)	LSIL	non-16/18 hr HPV	unifocal proximal 1/3	High-grade VaIN (VaIN 2)	Topical agent (imiquimod)	CR	24 months
Case 11	47	3/3	no	cervical cancer	yes (cervical cancer)	ASCUS	HPV 16	unifocal proximal 1/3	High-grade VaI (VaIN 2)	Topical agent (imiquimod)	PR	19 months
Case 12	63	4/3	yes	cervical cancer	yes (cervical cancer)	negative	non-16/18 hr HPV	N/A	High-grade VaIN (VaIN 3)	Topical agent (imiquimod)	CR	8 months *
Case 13	59	5/5	no	unknown	no	unknown	unknown	unifocal proximal 1/3	Low-grade VaIN (VaIN 1)	Observation	**	**
Case 14	52	1/1	no	cervical cancer	yes (cervical cancer)	ASCUS	non-16/18 hr HPV	multifocal proximal 1/3	Low-grade VaIN (VaIN 1)	Observation	CR	26 months
Case 15	37	4/4	no	cervical cancer	no	HSIL	non-16/18 hr HPV	multifocal proximal 2/3	High-grade VaIN (VaIN 3)	Topical agent (imiquimod)	CR	5 months *
Case 16	27	0/0	no	no	no	ASCUS	non-16/18 hr HPV	multifocal middle 1/3	High-grade VaIN (VaIN 2)	Surgical excision planned	No follow-up	
Case 17	51	4/1	no	no	yes (endometrial cancer)	negative	negative	unifocal distal 1/3	Low-grade VaIN (VaIN 1)	Observation planned	No follow-up	
Case 18	53	2/2	no	no	yes (endometrial cancer)	negative	negative	N/A	Low-grade VaIN (VaIN 1)	Observation	CR	18 months
Case 19	50	2/2	yes	no	yes (myoma uteri)	negative	HPV18	unifocal proximal 1/3	Low-grade VaIN (VaIN 1)	Topical agent (imiquimod)	CR	10 months
Case 20	47	3/2	no	CIN 3	yes (cervical dysplasia)	HSIL	HPV16	N/A	Low-grade VaIN (VaIN 1)	Topical agent (imiquimod)	Progression	14 months

Age: Age at VaIN diagnosis; G: Gravida; P: Parity; hr HPV: high-risk HPV; *: completed the treatment but were lost to follow-up after first visit; **: died of a malignant melanoma before the first follow-up visit; ^a^: initial treatment; ^b^: additional treatment; CR: complete response; PR: partial response; ASC-US: atypical squamous cells of undetermined significance; ASC-H: atypical squamous cells; cannot exclude a high-grade lesion; LSIL: low-grade squamous intraepithelial lesion; HSIL: high-grade squamous intraepithelial lesion.

**Table 2:** Treatment response and follow-up after imiquimod * in our case series.

Case	Age	Vaginal Biopsy and/or Cytology Before Imiquimod Treatment	HPV Status Before Imiquimod Treatment	Adverse Events	Treatment Period (Weeks)	First Vaginal Biopsy and/or Cytology After First Course Imiquimod Treatment	HPV Status After Imiquimod Treatment	Time Frame to NILM ** (Weeks or Months)	Response	RFI *** (Months)	Relaps
1	37	High-grade VaIN (VaIN 2/3)	negative	fever, pruritus	6 w/twice a week	Low-grade VaIN (VaIN 1)	negative	-	PR	N/A	N/A
2	61	High-grade VaIN (VaIN 2)	HPV 16	vaginal discharge	6 w/twice a week	NILM	non-16/18 hr HPV	11 m	PR	11 m	Low-grade VaIN
3	46	High-grade VaIN (VaIN 2/3)	non-16/18 hr HPV	none	6 w/twice a week	NILM	N/A	8 w	CR	11 m	Low-grade VaIN
4 ^1^	71	High-grade VaIN (VaIN 2/3)	non-16/18 hr HPV	none	8 w/twice a week	High-grade VaIN (VaIN 2/3)	non-16/18 hr HPV	24 m	CR	N/A	N/A
5	50	Low-grade VaIN (VaIN 1)	non-16/18 hr HPV	pruritus	8 w/twice a week	NILM	negative	11 m	CR	9 m	-
6	48	High-grade VaIN (VaIN 2/3)	non-16/18 hr HPV	none	12 w/twice a week	High-grade VaIN (VaIN 2/3)	N/A	24 m	CR	N/A	N/A
7	43	Low-grade VaIN (VaIN 1)	non-16/18 hr HPV	none	6 w/twice a week	NILM	negative	10 m	CR	N/A	N/A
10	43	High-grade VaIN (VaIN 2)	non-16/18 hr HPV	none	6 w/twice a week	NILM	negative	8 w	CR	22 m	-
11	47	High-grade VaIN (VaIN 2)	HPV 16	none	6 w/twice a week	Low-grade VaIN (VaIN 1)	non-16/18 hr HPV	-	PR	7 m	-
12	63	High-grade VaIN (VaIN 3)	non-16/18 hr HPV	none	6 w/twice a week	NILM	non-16/18 hr HPV	7 m	CR	N/A	N/A
15	37	High-grade VaIN (VaIN 3)	non-16/18 hr HPV	none	8 w/twice a week	NILM	N/A	4 m	CR	N/A	N/A
19	50	Low-grade VaIN (VaIN 1)	HPV 18	none	6 w/twice a week	Low-grade VaIN (VaIN 1)	negative	22 m	CR	N/A	N/A
20	47	Low-grade VaIN (VaIN 1)	HPV 16	pruritus	6 w/twice a week	High-grade VaIN (VaIN 3)	HPV 16	-	Progression	-	-

* A single sachet of 5% imiquimod cream (equivalent to 12.5 mg) to the vaginal lesions via a sterile swab, after inserting a speculum into the vagina; ^1^: Patient 4 received repeat imiquimode treatment following persistance after 6 months and 4 months; ** The median time to achieve a negative intraepithelial cytology (NILM: negative for intraepithelial lesion or malignancy) after starting the imiquimod; *** RFI, recurrence-free interval after clearence achieved; hr HPV: high-risk HPV; CR: complete response; PR: partial response.

**Table 3:** Summary of clinicopathologic data for patients.

	Low-Grade VaIN (*n* = 10)	High-Grade VaIN (*n* = 10)
Age (mean, min–max)	49.9 (34–60)	48.0 (27–71)
Gravidity (mean, min–max)	2.2 (0–5)	2.9 (0–7)
Parity (mean, min–max)	1.8 (0–5)	2.4 (0–6)
Smoking Status, *n* (%)		
Unknown	0	1 (10%)
Yes	3 (30%)	3 (30%)
No	7 (70%)	6 (60%)
Hysterectomy State, *n* (%)	8 (80%)	7 (70%)
Non-malignancy	4 (50%)	4 (57.1%)
Malignancy	4 (50%)	3 (42.9%)
Previous Cervical Neoplasia History, *n* (%)		
Unknown	1 (1%)	1 (1%)
Negative	3 (3%)	3 (3%)
CIN 1	1 (1%)	1 (1%)
CIN 2	0	0
CIN 3	3 (3%)	2 (2%)
Cervical cancer	2 (2%)	3 (3%)
Pre-Treatment PAP Smear Cytology, *n* (%)		
Unknown	1 (1%)	0
NILM	3 (3%)	4 (4%)
ASC-US	3 (3%)	2 (2%)
ASC-H	0	2 (2%)
LSIL	2 (2%)	1 (1%)
HSIL	1 (1%)	1 (1%)
Pre-Treatment HPV Status, *n* (%)		
Unknown	1 (1%)	0
negative	2 (2%)	1 (1%)
HPV 16	1 (1%)	2 (2%)
HPV 18	2 (2%)	0
Non 16/18 hr HPV	4 (4%)	7 (7%)
Treatment Modality, *n* (%)	*n* = 8	*n* = 9
Surgical excision	0	1/9 (1%)
Medical treatment (imiquimod)	4/8 (5%)	9/9 (10%)
Observation	4/8 (5%)	0
Post Medical Treatment Persistence, *n* (%)	*n* = 4	*n* = 9
Low-grade VaIN	0	3 (33.3%)
High-grade VaIN	1 (25%)	0
Response Rate to Medical Treatment		
Complete response	75%	66.7%
Partial response	0	33.3%

CIN: cervical intraepithelial neoplasia; NILM: negative for intraepithelial lesion or malignancy; ASCUS: atypical squamous cells of undetermined significance; ASC-H: atypical squamous cells, cannot exclude a high-grade lesion; LSIL: low-grade squamous intraepithelial lesion; HSIL: high-grade squamous intraepithelial lesion.

## Data Availability

The data that support the findings of this study are available on request from the corresponding author.

## References

[1] Watson M., Saraiya M., Wu X. (2009). Update of HPV-associated female genital cancers in the United States, 1999–2004. J. Womens Health.

[2] Sillman F.H., Fruchter R.G., Chen Y.S., Camilien L., Sedlis A., McTigue E. (1997). Vaginal intraepithelial neoplasia: Risk factors for persistence, recurrence, and invasion and its management. Am. J. Obstet. Gynecol..

[3] Suchonska B., Lugowski F., Papiez M., Ludwin A. (2025). Vaginal Intraepithelial Neoplasia (VaIN)-A Retrospective Cohort Analysis of Epidemiology, Risk Factors, and Management in an Academic Clinical Center. J. Clin. Med..

[4] Tranoulis A., Laios A., Mitsopoulos V., Lutchman-Singh K., Thomakos N. (2017). Efficacy of 5% imiquimod for the treatment of Vaginal intraepithelial neoplasia—A systematic review of the literature and a meta-analysis. Eur. J. Obstet. Gynecol. Reprod. Biol..

[5] Kim M.K., Lee I.H., Lee K.H. (2018). Clinical outcomes and risk of recurrence among patients with vaginal intraepithelial neoplasia: A comprehensive analysis of 576 cases. J. Gynecol. Oncol..

[6] Dodge J.A., Eltabbakh G.H., Mount S.L., Walker R.P., Morgan A. (2001). Clinical features and risk of recurrence among patients with vaginal intraepithelial neoplasia. Gynecol. Oncol..

[7] Rome R.M., England P.G. (2000). Management of vaginal intraepithelial neoplasia: A series of 132 cases with long-term follow-up. Int. J. Gynecol. Cancer.

[8] Hodeib M., Cohen J.G., Mehta S., Rimel B.J., Walsh C.S., Li A.J., Karlan B.Y., Cass I. (2016). Recurrence and risk of progression to lower genital tract malignancy in women with high grade VAIN. Gynecol. Oncol..

[9] Hummer W.K., Mussey E., Decker D.G., Dockerty M.B. (1970). Carcinoma in situ of the vagina. Am. J. Obstet. Gynecol..

[10] Darragh T.M., Colgan T.J., Cox J.T., Heller D.S., Henry M.R., Luff R.D., McCalmont T., Nayar R., Palefsky J.M., Stoler M.H. (2012). The Lower Anogenital Squamous Terminology Standardization Project for HPV-Associated Lesions: Background and consensus recommendations from the College of American Pathologists and the American Society for Colposcopy and Cervical Pathology. J. Low. Genit. Tract Dis..

[11] Sonnex C. (1994). Lower Genital Tract Precancer—Colposcopy, Pathology and Treatment. Genitourin. Med..

[12] Sherman J.F., Mount S.L., Evans M.F., Skelly J., Simmons-Arnold L., Eltabbakh G.H. (2008). Smoking increases the risk of high-grade vaginal intraepithelial neoplasia in women with oncogenic human papillomavirus. Gynecol. Oncol..

[13] Bruno M.T., Panella M.M., Valenti G., Di Grazia S., Sgalambro F., Farina J., Previti M., Mereu L. (2024). Vaginal Intraepithelial Neoplasia (VaIN) after Hysterectomy Is Strongly Associated with Persistent HR-HPV Infection. Cancers.

[14] Cao D., Wu D., Xu Y. (2021). Vaginal intraepithelial neoplasia in patients after total hysterectomy. Curr. Probl. Cancer.

[15] Imbertson L.M., Beaurline J.M., Couture A.M., Gibson S.J., Smith R.M., Miller R.L., Reiter M.J., Wagner T.L., Tomai M.A. (1998). Cytokine induction in hairless mouse and rat skin after topical application of the immune response modifiers imiquimod and S-28463. J. Investig. Dermatol..

[16] Kesic V., Carcopino X., Preti M., Vieira-Baptista P., Bevilacqua F., Bornstein J., Chargari C., Cruickshank M., Erzeneoglu E., Gallio N. (2023). The European Society of Gynaecological Oncology (ESGO), the International Society for the Study of Vulvovaginal Disease (ISSVD), the European College for the Study of Vulval Disease (ECSVD), and the European Federation for Colposcopy (EFC) consensus statement on the management of vaginal intraepithelial neoplasia. Int. J. Gynecol. Cancer.

[17] Silvestri F., Tosti G., Pepe F., Gandini S., Preti M. (2025). Off-Label Use of Imiquimod for Lower Female Genital Tract Diseases: A Systematic Review. J. Low. Genit. Tract Dis..

[18] Buck H.W., Guth K.J. (2003). Treatment of vaginal intraepithelial neoplasia (primarily low grade) with imiquimod 5% cream. J. Low. Genit. Tract Dis..

[19] Hendriks N., Koeneman M.M., van de Sande A.J.M., Penders C.G.J., Piek J.M.J., Kooreman L.F.S., Van Kuijk S.M., Hoosemans L., Sep S.J., Van Steenwijk P.J.D.V. (2022). Topical Imiquimod Treatment of High-grade Cervical Intraepithelial Neoplasia (TOPIC-3): A Nonrandomized Multicenter Study. J. Immunother..

[20] Trutnovsky G., Reich O., Joura E.A., Holter M., Ciresa-König A., Widschwendter A., Schauer C., Bogner G., Jan Z., Boandl A. (2022). Topical imiquimod versus surgery for vulvar intraepithelial neoplasia: A multicentre, randomised, phase 3, non-inferiority trial. Lancet.

[21] Inayama Y., Yamanishi Y., Nakatani E., Aratake J., Sasagasako N., Yamada K., Gou R., Kawamura A., Yamanishi M., Kosaka K. (2021). Imiquimod for vaginal intraepithelial neoplasia 2-3: A systematic review and meta-analysis. Gynecol. Oncol..

[22] Tainio K., Jakobsson M., Louvanto K., Kalliala I., Paavonen J., Nieminen P., Riska A. (2016). Randomised trial on treatment of vaginal intraepithelial neoplasia-Imiquimod, laser vaporisation and expectant management. Int. J. Cancer.

[23] Haidopoulos D., Diakomanolis E., Rodolakis A., Voulgaris Z., Vlachos G., Intsaklis A. (2005). Can local application of imiquimod cream be an alternative mode of therapy for patients with high-grade intraepithelial lesions of the vagina?. Int. J. Gynecol. Cancer.

[24] Field A., Bhagat N., Clark S., Speed T., Razvi K. (2020). Vaginal Intraepithelial Neoplasia: A Retrospective Study of Treatment and Outcomes Among a Cohort of UK Women. J. Low. Genit. Tract Dis..

